# Hydrocarbon-degrading bacteria in Colombia: systematic review

**DOI:** 10.1007/s10532-022-09976-z

**Published:** 2022-03-02

**Authors:** Diana Carolina Rache-Arce, Maryuris Machacado-Salas, Doris Rosero-García

**Affiliations:** Grupo de Investigación en Microbiología, Industria y Ambiente (GIMIA), Facultad de Ciencias Básicas, Universidad Santiago de Cali, Calle 5 # 62-00 Barrio Pampalinda, Santiago de Cali, Valle del Cauca, Colombia

**Keywords:** Bacteria, Colombia, Degradation, Hydrocarbons

## Abstract

Petroleum industry activities worldwide have caused pollution and resulted in environmental degradation. Microorganisms with the potential to reduce pollutant levels by degradation processes have been reported, and bacteria are among such organisms. The first study on bacterial degradation in Colombia was published in 1996. The study isolated bacteria belonging to the *Pseudomonas* genus from hydrocarbon-polluted sediments. Since then, different reports on degrading bacteria have been published. The objective of this systematic review is to identify and analyze all the studies on hydrocarbon-degrading bacteria performed in Colombia. To accomplish this goal, a literature search was conducted. Inclusion and exclusion criteria were applied, and 37 relevant articles were obtained. We found that 2018 was the year with the largest number of publications in Colombia, and most frequently identified bacterial genera were *Pseudomonas* and *Bacillus*. Some studies showed that the degradation of hydrocarbons is more efficient when bacterial consortia are used rather than pure cultures. This study provides information about bacteria with the potential to degrade hydrocarbons in Colombia, which in turn will be a source of information for future studies in this field.

## Introduction

Petroleum hydrocarbons are fossil fuels formed from organic matter; which are distributed in the subsoil layers and used for industrial energy production worldwide (Velásquez- Arias 2017). Currently, the presence of various kind of automobiles, the use of cleaning solvents, and some cosmetics may contain large amounts of hydrocarbons, which has caused an increase in their use (Ahmed and Fakhruddin 2018). The petroleum industry has grown in Colombia in recent years. The reserves of this fossil fuel are estimated to be about 1.5 billion barrels, which represents 26% of the country’s exports (Hernández-Rodríguez 2020). The growth of this industry has provided many benefits to the national economy by actively contributing to exports and the production of goods. The sector has further stimulated the generation of jobs and royalties for the financing of public expenditure (Hernández-Rodríguez 2020). However, unfortunately, petroleum sources also contribute to pollution and changes in land use as well as surface and groundwater utilization owing to exploitation, refining, lack of maintenance, and fuel theft (Sales da Silva et al. 2020). Moreover, Colombia has been affected by terrorist attacks approximately 829 times between 2007 and 2015 caused spills of thousands of barrels of hydrocarbons (Mendizabala et al. 2021). These problems may affect terrestrial and aquatic biodiversity due to landscape alteration (Sales da Silva et al. 2020).

In the abovementioned context, microorganisms with the potential to reduce pollutant levels by degradation processes have gained attention (Garzón et al. 2017; Sales da Silva et al. 2020). Bacteria are among those microorganisms that are able to convert the pollutants to less toxic molecules, and hence, allow the reclamation of large expanses of polluted areas (Hernández Ruiz et al. 2017; Renteria and Rosero 2019). Bacteria are capable of tolerating and using certain pollutants as sources of carbon and energy, contributing to the remediation of affected ecosystems (Marquez-Rocha et al. 2001). The oxygen-dependent enzymes called monooxygenases provide a means to use hydrocarbons as substrates, which allows the survival of bacteria in hydrocarbon-polluted environments (Das and Chandran 2011). Certain bacteria isolates such as *Escherichia coli*, *Alcaligene*s sp. and *Thiobacter subterraneus* can contribute in the degradation process by combining several metabolic pathways in a consortium to increase the extent of degradation of polycyclic aromatics hydrocarbons-PAHs (Pandey and Dubey 2012). Another important aspect is the presence of indigenous bacterial populations, which are of interest in degradation studies as they can be directly isolated from polluted sites and be characterized for a better understanding of the mechanism of biodegradation (Das and Chandran 2011).

The first study on bacterial degradation in Colombia, published in 1996, isolated bacteria belonging to the *Pseudomonas* genus from sediments highly polluted by PAHs (Vargas et al. 1996). Since then, several studies have been published, including reviews that list hydrocarbon-degrading bacteria (HDB) and discuss the importance of their management in polluted environments (Lozano 2005; Benavides-López et al. 2006; Trujillo-Toro and Ramírez-Quirama 2012; Garzón et al. 2017; De La Rosa Martinez and Rabelo-Florez 2020). However, thus far, there is no known review gathering data from all the research on hydrocarbon-degrading bacteria, advantages, and applications in Colombia. Since the problem of hydrocarbon pollution is of global relevance (Zhang and Chen 2017; Sales da Silva et al. 2020) and Colombia also considers it a critical issue. Therefore, in this review the objective is to identify all the studies on HDB conducted in the country so far. This paper provides an analysis about bacterial hydrocarbon degradation capability, pinpoint the areas in which degradation studies have been performed, and identify the most evaluated hydrocarbon. This information towards the better understanding in bioremediation challenges and will allow researchers interested in this field to have adequate baseline information to plan future studies.

## Materials and methods

Investigations were selected from the Scielo, PubMed, Redalyc, ScienceDirect, Scopus, and Dialnet databases. Google Scholar was also used for the search of gray literature, and for peer reviewed articles. The following keywords were defined in Spanish (degradación, Colombia, hidrocarburos, bacterias), and in English (degradation, Colombia, hydrocarbon, bacteria). Different combinations of last keywords were employed to obtain a high number of publications in the exhaustive search. For the selection of publications suitable for analysis, the following inclusion criteria were established: type of study (original articles and theses), place (Colombia), degraded pollutant (petroleum, diesel, gasoline, motor oil), degrading microorganism (bacteria), publication date (between 1996 and 2021), and language (Spanish and English). During the literature search, those articles that did not meet the established criteria were excluded: articles about studies performed outside Colombia, degrading organisms other than bacteria, such as fungi, microalgae, and plants, and degraded pollutants other than hydrocarbons, such as heavy metals and pesticides.

The results of the analysis of the collected studies were recorded in a table using Microsoft® Excel 2019 according to author’s name, year of publication, source in which the study was conducted, type of study, and identified bacterium (genus and/or species). Additionally, an analysis to determine the behavior and the interest in studying HDB between 1996 and 2021 was performed. The impact and the interest in research on this topic in Colombia were assessed and compared with some reviews performed for other regions of the world.

## Results

The exhaustive search yielded 1288 articles, 410 of which were published in Spanish and 878 in English (Table 1).

**Table 1:** General statistical information for the articles of hydrocarbon-degrading bacteria (until May 2021)

Database	Spanish	English
Number of articles found in the search results by database		
Scielo	59	154
PubMed	0	82
Redalyc	0	137
ScienceDirect	1	302
Scopus	0	0
Dialnet	0	1
Google Scholar	350	202
Total number of articles	410	878

**Fig. 1:**
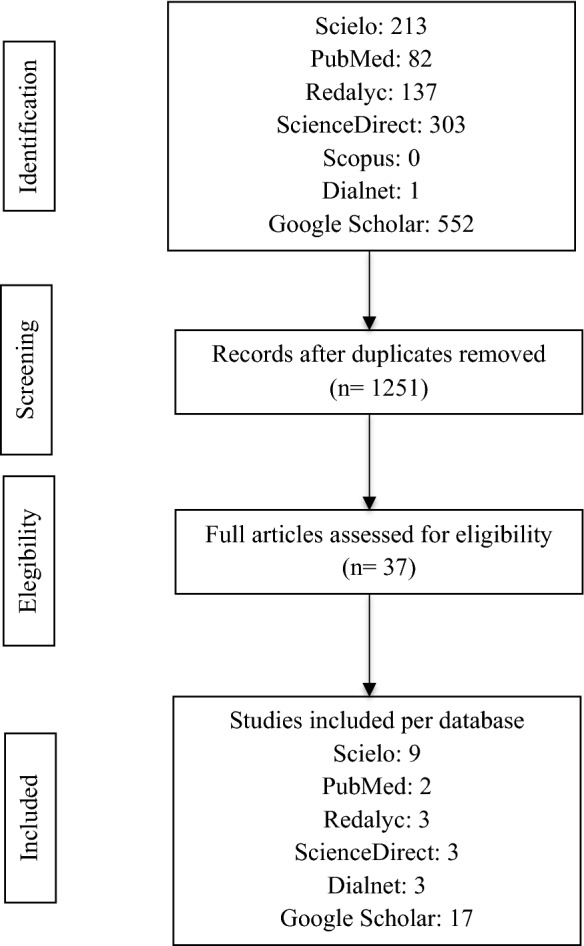
PRISMA flowchart showing the selection of the research articles of hydrocarbon-degrading bacteria in Colombia

After removal of the duplicate articles and application of the inclusion criteria, 37 articles were obtained (Fig. 1).

**Fig. 2:**
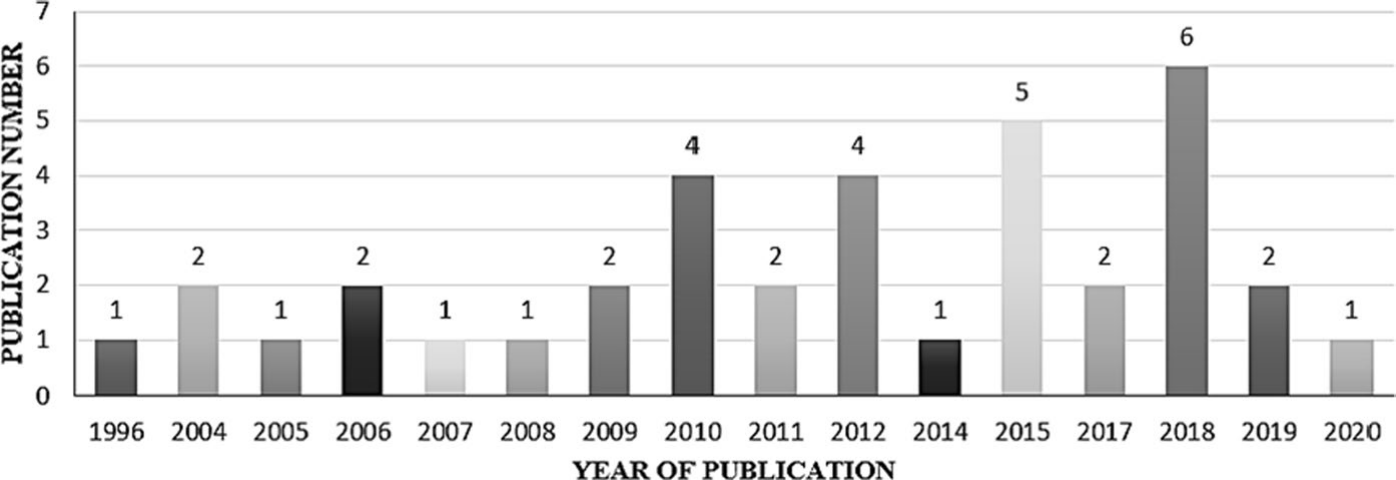
Number of studies on HDB in Colombia between 1996 and 2020

**Fig. 3:**
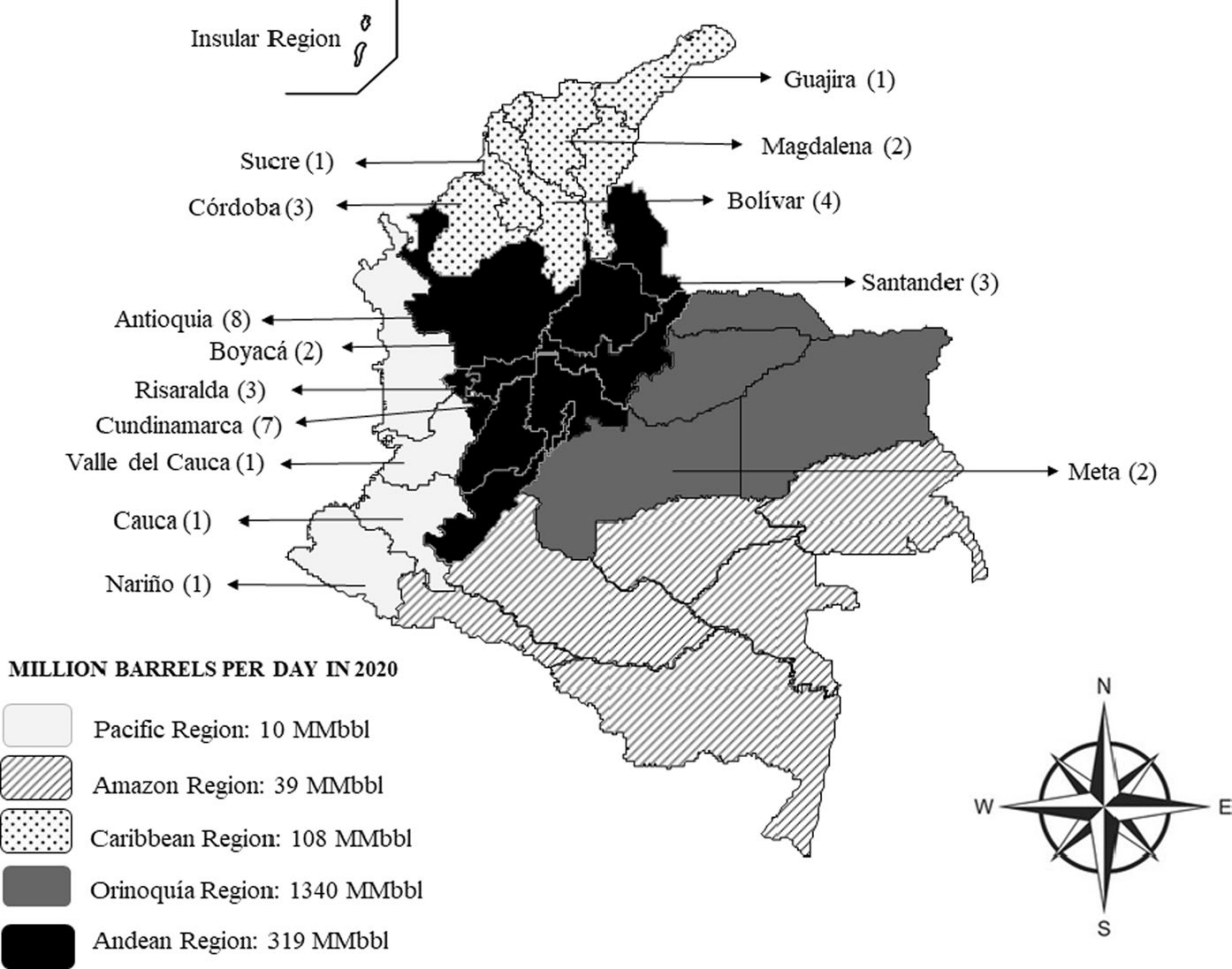
Map of Colombia showing the number of publications per department and oil production by region

From the analysis of the 37 selected publications, it was observed that a high number of studies on bacterial hydrocarbon degradation were published mainly in 2018 (Fig. 2). This study was done in Colombia’s subnational territories, which comprise Bogotá as Capital District (C.D.), and 32 political-administrative entities called departments. Moreover, the country is divided into six natural regions constituted by differences in topography, weather, vegetation, types of soil and oil production. The Andean Region, covering the three branches of the Andes mountains; the Caribbean Region, covering the area adjacent to the Caribbean Sea; the Pacific Region adjacent to the Pacific Ocean; the Orinoquía Region, part of the Llanos plains mainly in the Orinoco River basin along the border with Venezuela; the Amazon Region, part of the Amazon rainforest; and finally the Insular Region, comprising islands in both the Atlantic and Pacific oceans (Fig. 3). Among the departments in which a high number of studies on HDB have been conducted are Antioquia and Cundinamarca with eight and seven publications respectively, located at Andean Region with a oil production of 319 Million barrels per day in 2020 (Minenergía 2021). Remarkably, the Orinoquía Region had the highest oil production, but two studies have been conducted only (Fig. 3).

**Fig. 4:**
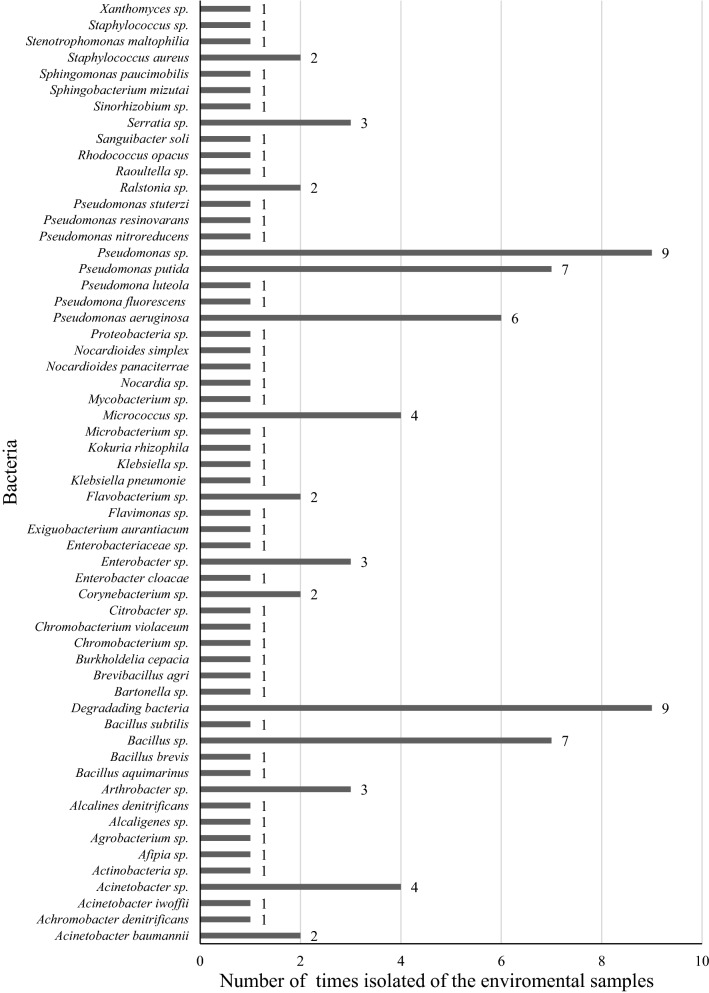
Hydrocarbon degrading bacteria (HDB) in Colombia and their frequency in the analyzed publications

*Pseudomonas* sp. was the most representative genus of HDB on the papers in Colombia (Table 2). In the present study, 19 publications describing the isolation of bacteria belonging to this genus with *Pseudomonas aeruginosa* and *Pseudomonas putida* being the most frequently isolated species (Fig. 4). Furthermore, 7 publications reported bacteria belonging to the *Bacillus* genus (Table 2). Some studies did not report the bacterial genus and/or species because unidentified strains from bacterial consortia were used (Table 2). Among the analyzed studies, 16 on petroleum, 12 on diesel, 4 on gasoline, 1 on oil motor, 1 on kerosene, and 1 on tar. Three studies did not report the evaluated hydrocarbon (Table 2). In addition, an analysis of the universities, companies, and research groups that participated in the publications was performed (Table 3). The bacterial strains able to degrade hydrocarbons were isolated and identified, from soils samples mainly (Table 2).

**Table 2:** Studies of hydrocarbon degrading bacteria conducted in Colombia

Authors and year of publication	Source of the environmental samples					Hydrocarbon-degrading bacteria (HDB) isolated/identified	Evaluated hydrocarbon
	Place	Department	Type of study	Environmental sample	Methodology for hydrocarbon-degrading bacteria isolation/identification		
Vargas et al. (1996)	Bucaramanga	Santander	Original article	Contaminated soils	Two selection systems called fast route and slow route	*Pseudomonas stutzeri*, *Pseudomonas aeruginosa*, *Pseudomonas resinovarans*, *Pseudomonas nitroreducens*	Petroleum
Suárez-Medellin and Vives (2004)	Bogotá D.C	Cundinamarca	Master’s thesis	Contaminated soils	Direct isolation/traditional microbiology	*Pseudomonas luteola*, *Pseudomonas putida*, *Micrococcus* sp.*, Alcalines denitrificans*, *Pseudomonas* sp., *Pseudomonas aeruginosa*	Gasoline
Perdomo-Rojas and Pardo-Castro (2004)	Zipaquirá	Cundinamarca	Undergraduate thesis	Contaminated soils	Direct isolation/BBL™ Crystal™ Identification Systems	Degrading bacteria: gram-positive and gram-negative bacilli	Petroleum
Vallejo et al. (2005)	Bogotá D.C	Cundinamarca	Original article	Contaminated soils	Direct isolation/biochemical test	*Stenotrophomonas maltophilia*, *Acinetobacter iwoffii*, *Burkholderia cepacia*, *Pseudomonas putida, Chomobacterium violaceum, Flavimonas oryzihabitants*	Petroleum
Gomez et al. (2006)	Colombian Caribbean	Bolívar, Córdoba, Magdalena, Sucre	Original article	Sediments	Direct isolation/Strains were identified by 16S rRNA	*Klebsiella pneumoniae, Enterobacteriaceae bacterium, Pseudomonas* sp.*, Ralstonia* sp.*, Bacillus pumilus*, *Acinetobacter* sp.*, Brevibacillus agri*	Petroleum
Duran-Rincon and Contreras (2006)	Pereira	Risaralda	Original article	Soils	Direct isolation	*Arthrobacter* sp., *Bacillus* sp., *Pseudomonas* sp., *Agrobacterium* sp*.*, *Alcaligenes* sp., *Flavobacterium* sp., *Corynebacterium* sp., *Micrococcus* sp*.*, *Taphylococcus* sp., *Xanthomonas* sp*.*, *Mycobacterium* sp.	Diesel
Camargo-Millán and Acero-Pérez (2007)	Tunja	Boyacá	Original article	Contaminated soils	Inoculation with *Pseudomonas aeruginosa* bacteria	*Pseudomonas aeruginosa*	Petroleum
Narváez-Flórez et al. (2008)	Colombian Caribbean	Bolívar, Córdoba, Magdalena, Sucre	Original article	Sediments	Direct isolation/BBL crystal and API 50 CHB/E	*Klebsiella* sp., *Chromobacterium* sp., *Flavimonas orizihabitans*, *Enterobacter cloacae, Pseudomonas aeruginosa, Bacillus brevis, B. pumillus, B. cereus,*	Diesel, petroleum
Kopytko and Ibarra-Mojica (2009)	Bucaramanga	Santander	Original article	Soils	Direct isolation	*Serratia* sp.	Petroleum
Gómez et al. (2009)	Medellín	Antioquia	Original article	Soils	Direct isolation	*Bacillus* sp*.*	Diesel, gasoline
Nisperuza-Vidal and Montiel-Aroca (2010)	San Sebastián	Córdoba	Undergraduate thesis	Crude oil well	Direct isolation/Api20E®, Api20NE® and the software ApiWeb®	*Burkholdelia cepacia*, *Pseudomonas putida*, *Pseudomonas fluorescens*, *Pseudomonas aeruginosa*	Petroleum
Vásquez et al. (2010)	Río Frío	Santander	Original article	Sludge	Direct isolation/Biochemical test and BBL CRYSTAL-NF	*Pseudomonas* spp., *Acinetobacter* spp*.*, *Enterobacter cloacae*, *Citrobacter* spp*.*, *Bacillus brevis*, *Micrococcus* spp., *Nocardia* spp.	Diesel
Yanine (2010)	Complejo Ecorregional Andes del Norte (CEAN), Pereira	Risaralda	Master’s thesis	Soils	Direct isolation/Strains were identified by 16S rRNA	49 degrading bacteria species (See reference for list)	Diesel
Vallejo et al. (2010)	Ecoregión cafeteria	Valle, Risaralda, Quindío	Original article	Soils	Direct isolation/Inoculation with *Acinetobacter* sp. bacteria	Degrading bacteria: gram-positive	No data available
Echeverri Jaramillo et al. (2011)	Cartagena	Bolívar	Original article	Biofilms, sediment, or sludge, neuston and water subsurface	Direct isolation/Biochemical test	*Pseudomonas aeruginosa*	Petroleum
García et al. (2011)	Bogotá D.C	Cundinamarca	Original article	Contaminated soils	Direct isolation	Degrading bacteria	Petroleum
Arrieta-Ramírez et al. (2012)	Medellín	Antioquia	Original article	Soils	Direct isolation/Strains were identified by 16S rRNA	*Enterobacter* sp., *Bacillus* sp., *Staphylococcus aureus*, *Sanguibacter soli*, *Arthrobacter* sp., *Flavobacterium* sp.	Diesel
Pino et al. (2012)	Apartadó	Antioquia	Original article	Soils	Direct isolation	Degrading bacteria	Diesel
Quintana-Saavedra et al. (2012)	Cartagena	Bolívar	Original article	Water	Direct isolation/Biochemical test	*Pseudomonas* sp.*,* *Bacillus subtilis, Staphylococcus* sp.	Diesel, gasoline
Gómez-Rivera and Kopitko (2012)	Puerto Boyacá	Boyacá	Undergraduate thesis	Soils	Direct isolation	*Pseudomonas* spp.	Petroleum
Ñuste-Cuartas et al. (2014)	Dosquebradas	Risaralda	Original article	Sewage water	Direct isolation	Degrading bacteria	Diesel, gasoline
Pérez-Robles et al. (2015)	Medellín	Antioquia	Original article	Soils	No data available	Degrading bacteria	Diesel, gasoline
Barrios-Ziolo et al. (2015)	Medellín	Antioquia	Original article	Soil contaminated with used motor oils	Direct isolation/Traditional microbiology	Coccus and bacilli gram-negatives	Oil motor
Mezquida et al. (2015)	Lorica	Córdoba	Original article	Soil	Direct isolation/ macroscopic and microscopic observations, biochemical tests. Commercial kits Api20E® and Api20NE®	*Achromobacter denitrificans*, *Sphingomonas paucimobilis*, *Pseudomonas putida, Brevundimonas vesicularis, Acinetobacter baumanii, Rhizobium radiobacter, Comamonas testosteroni, Chryseobacterium indologenes*	Diesel
Vallejo-Quintero et al. (2016)	Soacha	Cundinamarca	Original article	Soils	Direct isolation	Degrading bacteria	Not data reported
Álvares et al. (2016)	Medellín	Antioquia	Undergraduate thesis	Water	Direct isolation/Biochemical test, VITEK®	*Pseudomonas* sp., *Serratia* sp*., Bacillus* sp.	Tar
Pardo-Díaz et al. (2017)	Castilla la Nueva y Apiay	Meta	Original article	Soils	Direct isolation/Strains were identified by 16S rRNA	*Pseudomonas* sp., *Pseudomonas putida*, *Achromobacter* sp.	Petroleum
Delgado-Vallejo (2017)	Medellín	Antioquia	Master’s thesis	Soils	No data available	Degrading bacteria	Petroleum
Ordoñez-Burbano et al. (2018)	Cali	Valle del Cauca	Original article	Soils	Direct isolation/ BBL CRYSTAL™	*Burkholderia cepacia*	Kerosene
Doria-Argumedo (2018)	Rioacha	La Guajira	Original article	Soils	Direct isolation	*Pseudomonas* spp*., Acinetobacter* spp*.*, *Bacillus* spp*.*	Diesel
Martínez-Rivera (2018)	Medellín	Antioquia	Master’s thesis	Soils	Direct isolation/Metagenomic V3-V4 region/ 16S rRNA	21 degrading bacteria Phyla (See reference for list)	Petroleum
Malaver and Muñoz (2018)	Cajibio	Cauca	Undergraduate thesis	Soils	No data available	Degrading bacteria	Petroleum
Reyes-Reyes et al. (2018)	Región centro-oriental	Campo petrolero	Original article	Sludges	Direct isolation/Strains were identified by 16S rRNA	*Bacillus* sp*., Pseudomonas* sp*.*, *Serratia* sp., *Raoultella* sp*.*, *Enterobactr* sp.	Petroleum
Pinto-Varón and Sánchez-Vargas (2018)	Bogotá D.C	Cundinamarca	Undergraduate thesis	Soils	Inoculation with two bacteria	*Pseudomona putida*, *Acinetobacter baumannii*	Diesel, gasoline
Galvis-Ibarra (2019)	San Carlos de Guaroa	Meta	Undergraduate thesis	Oily sludge (Oil residue)	Inoculation with consortium bacteria	Degrading bacteria	Petroleum
Garcés-Ordoñez and Espinoza-Díaz (2019)	Mira river, Tumaco	Nariño	Original article	Mangrove sediments	No data available	Degrading bacteria	Not data reported
Arenas-Soler (2020)	Bogotá, D.C	Cundinamarca	Undergraduate thesis	Bioassays	Inoculation with bacteria	*Chromobacterium violaceum*, *Pseudomonas aeruginosa*	Diesel

**Table 3:** Universities, companies, and research groups that have published on Hydrocarbon Degrading Bacteria (HDB) in Colombia

Universities and companies	Research groups or laboratories	#	Authors and year of publication
Universidad Nacional de Colombia	Biorremediación y Desarrollo Tecnológico	2	Delgado-Vallejo (2017); Martínez-Rivera (2018)
	Grupo de investigación en Ciencias de los Alimentos	1	Arrieta-Ramírez et al. (2012)
	PARH-Posgrado de Aprovechamiento de Recursos Hidráulicos	1	Pérez-Robles et al. (2015)
	Laboratorios de Química de Suelos, Análisis Instrumental, Microbiología Molecular y Microbiología Industrial / Laboratorio de Hidráulica/Laboratorio de Microbiología Ambiental y Aplicada	3	Gómez et al. (2009); Barrios-Ziolo et al. (2015); Pardo-Díaz et al. (2017)
	CIEBREG-Centro de Investigaciones y Estudios en Biodiversidad y Recursos Genéticos	1	Yanine (2010)
Universidad Pedagógica y Tecnológica de Colombia	GIGA-Grupo de Investigación en Geomática y Ambiente	1	Camargo-Millán and Acero-Pérez (2007)
Universidad de La Guajira	Grupo de Investigación Territorios Semiáridos del Caribe	1	Doria-Argumedo (2018)
Universidad de los Andes	CIMIC-Centro de Investigaciones Microbiológicas	2	Suárez-Medellin and Vives (2004); Gomez et al. (2006)
Universidad de San Buenaventura	GIMA-Grupo de Microbiología y Ambiente	1	Echeverri Jaramillo et al. (2011)
	CIOH-Centro de Investigaciones Oceanográficas e Hidrográficas del Caribe	1	Quintana-Saavedra et al. (2012)
Universidad de Antioquia	GDCON-Diagnostic and Pollution Control Group	1	Pino et al. (2012)
Universidad de La Salle	Laboratorios de Microbiología de la Universidad de La Salle	2	Perdomo-Rojas and Pardo-Castro (2004); Arenas-Soler (2020)
Universidad Tecnológica de Pereira	Agua y Saneamiento	1	Ñuste-Cuartas et al. (2014)
	Laboratorio de Oleoquímica de la escuela de Química	2	Duran-Rincon and Contreras (2006)
Pontificia Universidad Javeriana	USBA-Unidad de Saneamiento y Biotecnología Ambiental	5	Vallejo et al. (2005); García et al. (2011); Vallejo-Quintero et al. (2016); Pardo-Díaz et al. (2017); Galvis-Ibarra (2019)
	A.T.P Ingeniería S.A.S	1	Galvis-Ibarra (2019)
Universidad Pontifica Bolivariana	Centro de Investigación en Biotecnología, Biotécnica y Ambiente	1	Kopytko and Ibarra-Mojica (2009)
	SINSA	1	Gómez-Rivera and Kopitko (2012)
Universidad de Córdoba	GRUBIODEQ-Grupo de Investigación en Biotecnología	2	Nisperuza-Vidal and Montiel-Aroca (2010); Mezquida et al. (2015)
Universidad del Valle	Laboratorio de Docencia de Microbiología de la Universidad del Valle	1	Ordoñez-Burbano et al. (2018)
Universidad Libre	Laboratorios de Ingeniería Ambiental de la Universidad Libre	1	Pinto-Varón and Sánchez-Vargas (2018)
Universidad de Santander	Laboratorio Clínico de la Universidad de Santander (UDES)	1	Vásquez et al. (2010)
Fundación Universidad de America	Not information available	1	Arenas-Piza (2018)
Universidad Industrial de Santander	Corporación para la Investigación de la Corrosión	1	Reyes-Reyes et al. (2018)
Universidad central de Colombia	Agua y Desarrollo Sostenible	1	Gamba and Pedraza (2017)
Institución universitaria colegio mayor de Antioquia	Biociencias	1	Álvarez-Mejia et al. (2016)
Corporación Universitaria Autónoma del Cauca	Laboratorio de la Facultad Ciencias ambientales y Desarrollo Sostenible	1	Malaver and Muñoz (2018)
Instituto de Investigaciones Marinas y Costeras IVEMAR	Laboratorios de Calidad Ambiental Marina	1	Garcés-Ordoñez and Espinoza-Díaz (2019)
	Programa Calidad Ambiental Marina	1	Narváez-Flórez et al. (2008)
Fundación Universitaria Tecnológico Comfenalco	GIA-Grupo de Investigaciones Ambientales	1	Echeverri Jaramillo et al. (2011)
Ecopetrol—Instituto Colombiano del Petróleo	Not information available	1	Vargas et al. (1996)

## Discussion

This systematic review was designed to provide the most complete, up-to-date list of studies about hydrocarbon-degrading bacteria (HDB) in Colombia, with a total of 37 investigations. Selecting the HDB is of profound significance in evaluating, developing, and designing strategies for bioremediation studies owing to their potential to adapt to polluted environments and convert the pollutants such as hydrocarbons to innocuous substances by degradation (Das and Chandran 2011). Moreover, it is important to perform studies to identify bacteria with degradation capability like an important step toward successful bioremediation (Reyes-Reyes et al. 2018). In the present review, we found that authors from different universities, companies, and research groups have conducted studies in Colombia to isolate HDB on environmental samples since 1996 (Vargas et al. 1996). For Colombia, 2003 was a year of substantial advances with regard to petroleum exploration given that reforms attracted foreign investment (Trujillo-Quintero et al. 2017). Probably, this is the reason for an increase in publications after 2003. Most of the publications were from 2018, it is likely that the above issue might have aroused the interest of different researchers to study microbial degradation and provide possible solutions for the pollution problem using bioremediation (Renteria and Rosero 2019). Moreover, the increasing available grants to investigations and doctoral formation in last year’s support the results obtained here (Minciencias 2019).

In the Andean Region, the departments of Antioquia and Cundinamarca, there are research groups in microbiology, chemical engineering, and biotechnology, among others. This observation emphasizes the fact that this region is very much interested in and at the cutting edge of studies in HDB. Concerning the research groups, the ones belonging to Universidad Nacional de Colombia, particularly in Medellín at Antioquia department, and Pontificia Universidad Javeriana in Bogotá, D.C. at Cundinamarca department reported the highest number of publications on HDB in Colombia. This establishes the need to continue the search for HDB in all the departments of the country, mainly in those located in regions with high oil production where can provide hydrocarbon residues.

The occurrence of some species belonging to the *Pseudomonas* and *Bacillus* genera and others mentioned here, constitutes valuable information for HDB present in Colombia. According to the analyzed publications, *Pseudomonas* and *Bacillus* species are the most frequently isolated in hydrocarbon degradation studies in the country. Probably, this result could be attributed to the much higher cultivability of both genera by direct isolation of contaminated samples with hydrocarbons (Gomez et al. 2006; Quintana-Saavedra et al. 2012; Álvares et al. 2016; Doria-Argumedo 2018). However, *Pseudomonas* and *Bacillus* are genera truly important and have been found to play vital roles in petroleum hydrocarbon degradation (Vásquez et al. 2010; Yanine 2010; Das and Chandran 2011; Xu et al. 2018). For example, *P. aeruginosa* has been identified as a HDB capable of degrading aromatic and polyaromatic hydrocarbons because it produces biosurfactants during its stationery growth phase, which facilitates the solubilization and therefore the degradation (Silva et al. 2018). Inoculation with *P. aeruginosa* bacteria had the highest rates of hydrocarbon removal, in ground contaminated samples with the Castilla`s crude, coming from 10 fields (Camargo-Millán and Acero-Pérez 2007). *Pseudomonas putida* is part of the soil microbiota and possesses enzymes called dioxygenases that are involved in hydrocarbon degradation (Truskewycz et al. 2019). On the other hand, species belonging to the *Bacillus* genus present high adaptability and can grow in extreme and hostile environments such as hydrocarbon contaminated soil and water (de Mesa et al. 2006; Valdivia-Anistro et al. 2018). Furthermore, the *Bacillus* genus is another bacterium reported as petroleum hydrocarbon degrader, and could be useful in reducing the levels of these hydrocarbons (Kolsal et al. 2017; Lima et al. 2020).

Another important result is taxonomic information for some HDB is unknown (Perdomo-Rojas and Pardo-Castro 2004; Vallejo et al. 2010; Yanine 2010; García et al. 2011). Additional investigations using molecular and other tools to identify all HDB is highly desirable in these cases. Overall, the taxonomy of environmental bacteria in Colombia is relatively poorly known. The taxonomic category of HDB is important for planning and interpreting future biodegradation studies (Ławniczak et al. 2020). In addition, the degradation of hydrocarbons is more effective when bacteria work together in a consortium. For example, Arrieta et al. showed the efficiency of a bacterial consortium that included the genera *Arthrobacter*, *Bacillus*, *Flavobacterium*, *Sanguibacter*, and *Staphylococcus* in the degradation of diesel (Arrieta-Ramírez et al. 2012). Vásquez et al*.* used a bacterial consortium composed of *Acinetobacter, Bacillus brevis, Citrobacter*, *Enterobacter cloacae*, *Micrococcus*, *Nocardia*, and *Pseudomonas* to study the degradation of oil sludge from a car wash (Vásquez et al. 2010). The hydrocarbons evaluated in the 37 selected publications; petroleum was the most studied one. In general, the authors suggest that short-chain aliphatic hydrocarbons such as those found in gasoline are more likely to volatilize and also tend to be toxic for bacteria (Suárez-Medellin and Vives 2004; Narváez-Flórez et al. 2008). This fact could explain why there are not as many studies on gasoline degradation as on petroleum.

This systematic review addresses studies specifically performed in Columbia with Columbian environmental samples. A more thorough investigation of knowledge about HDBs in different regions and their role in bioremediation of contaminated sites is useful. There are few similar studies that have systematically reviewed HDBs identified from specific regions or countries around the world. A review of remediation approaches for petroleum hydrocarbon contamination in the Arctic and Antarctic regions included bioremediation and identified bacteria isolated from these regions (Camenzuli and Freidman 2015). A recent review of PAH contamination in China, a country where rapid industrialization and urbanization have created fast economic growth, focused more on sources of PAHs in soils, but not on biodegradation (Zhang and Chen 2017). Other recent reviews examined more generally petroleum hydrocarbon biodegradation in aquifers (Logeshwaran et al. 2018) and provided an overview of enhanced hydrocarbon biodegradation strategies (Ławniczak et al. 2020). Notably, in Colombia a review article provided information regarding the most representative bacterium in biodegrading hydrocarbons *Pseudomonas* sp., *Bacillus* sp., *Bacillus subtilis* and *Burkholderia sp.* (De La Rosa Martinez and Rabelo-Florez 2020). A compilation of investigations conducted inside a specific country is important for establishing a baseline and needs for future research. This is especially pertinent in countries such as Colombia due to the presence of hydrocarbons as substantial contaminants in different ecosystems throughout the country, and where much research is still needed. We considering that it is also important that similar systematic reviews be conducted by researchers in the different countries to know the HDB and the studies that may be required to control hydrocarbons contamination.

## Conclusions

A literature search yielded 1288 articles on HDB. After applying the inclusion criteria, 37 published studies were identified in Colombia between 1996 and 2020. However, among these, no doctoral theses were found. Most of the publications were from 2018, and *Bacillus* sp. and *Pseudomonas* sp. are the most studied genera in Colombia. Particularly, *P. aeruginosa* and *P. putida* are the most assessed species owing to the metabolic variation and enzymatic production that allow them to adapt to environments polluted with hydrocarbons. It was observed in several studies that hydrocarbon degradation is more efficient when bacterial consortia are used rather than pure cultures. The most studied hydrocarbon in Colombia is petroleum, while the least reported ones are oil motor, kerosene, and tar. Finally, this study is important because it provides useful information about bacteria that exhibit the potential to degrade hydrocarbons in Colombia.

## Data Availability

Not applicable.
